# A New Approach for Constructing a Health Care Index including the Subjective Level

**DOI:** 10.3390/ijerph19159686

**Published:** 2022-08-05

**Authors:** Sandra Jaworeck

**Affiliations:** 1 Institute of Sociology, Chemnitz University of Technology, 09107 Chemnitz, Germany; sandra.jaworeck@hsw.tu-chemnitz.de; 2 Institute of Labor Sciences, Ruhr-University Bochum, 44801 Bochum, Germany

**Keywords:** health care systems, index, subjective, objective, micro, macro

## Abstract

Until now, health care systems have been compared by means of macro criteria, an approach that might have its shortcomings in assessing the actual benefits that health care systems may provide for people. Therefore, a new health care index is presented which combines individual assessments of health care systems with objective macro health care system criteria. Two steps are taken for furthering this approach: First, a data-driven procedure is used to determine the influence of self-rated health on confidence in the health care system through macro criteria of health care systems. The macro indicators are weighted accordingly and created into an index, which adds to the subjective level of the link. In a second step, the constructed health care index is tested in a multilevel model with self-rated health being the dependent variable, to avoid tautological conclusions. The index is able to reduce country differences, decrease explained variability and has a statistically significant effect without affecting other estimates.

## 1. Introduction

In most countries of the world, there is an understanding that the state should provide adequate health care for the population. However, health care systems are complex and differ greatly between countries [1]. Originally, health care systems are divided into tax-financed care networks (Beveridge) and care that is based on the (social) insurance system (Bismarck) [2].

The Beveridge model is built on a central supply principle of the state and financed by taxes. It is a public provision of services for the entire population and implemented for example in Denmark, Finland, Great Britain, Italy, Spain, Norway, Portugal and Sweden [2]. The Bismarck model is based on health insurance financed by social contributions from the insured and their employers. It is implemented in countries such as France, Belgium, Luxembourg, Japan, Austria, Germany, Netherlands and Switzerland. Comparing them with each other is and always has been a particular challenge [2]. This original classification dates back a long time. In the meantime, much has changed. Yet the interest in and importance of comparing health care systems is accounted for by the amount of already existing health care system indices.

The Euro Health Consumer Index (EHCI), for example, covers five dimensions: patient rights and information, waiting times for common treatments, success of treatment and generosity of the system, as well as access to medication [3]. Another example, the Global Health Security Index (GHSI) attempts to provide a global overview of health care systems by measuring, among other things, their resilience. It operationalizes indicators such as staff protection, number of staff, hospital facilities and proportion of people with access to the health care system [4]. Further, the Future Health Index examines how decision makers are meeting today’s health care demands [4]. In addition, the Kakwani Index measures the equitable financing of health care [5]. Lastly, the Bloomberg Global Health Index looks at a variety of factors that contribute to a country being considered healthy or unhealthy, for example, low pollution rates, access to quality health care and clean drinking water but also health risks such as smoking, hypertension, obesity, life expectancy, malnutrition and causes of death [6]. It would be possible to write a whole book just about different health indices.

These indices, although covering a wide range of indicators, all have one thing in common: they include exclusively objective macro criteria. By objective macro indicators, it is meant that something can be measured in an unbiased way and aggregated statistic indicates how well this is done from country to country. The shortcomings of these kinds of measures become clear when looking at a case study such as the United States of America (U.S.). The U.S. is highly advanced in terms of medical research, while there is at the same time only limited access to the health care system. In reality, therefore, very few residents can take advantage of the resources (subjective access on micro level). The U.S. case study is a good example for the fact that an aggregate measure in itself does not allow for inferences about the individual level, a statistical condition which when neglected is also known as an ecological fallacy [7]. In order to get a full picture of the situation within a country and considering that populations have different access requirements to health care systems, it is important to capture the individual assessment of residents on access to their health care system as well.

Hence, I suggest that when comparing health care systems, it is not only important to include objective macro criteria, but also to evaluate access to the health care system and the actual benefits it provides for citizens. In other words, the focus from a sociological perspective should not only be on the hypothetical availability of resources as indicated by objective macro criteria but rather on the actual well-being of people.

Therefore, I present a new health care index which combines people’s individual assessment of national health care systems with objective macro health care system criteria. Against the backdrop of the previous considerations, my research question for creating the new index is: How are people actually doing in their country?

Is the quality of a health care system more dependent upon the hypothetical availability of resources or upon what people in reality have access to? Needless to say, for a country comparison the objective macro criteria must not be ignored. They play an important role. Therefore, it is of great interest to combine both levels and to look at them simultaneously.

For this purpose, the impact of self-rated health on confidence in the health care system is considered and explored using established objective macro indicators of health care systems. The objective criteria are then weighted according to subjective relevance and combined to form a new index. How this is done is described in Section 2.3.2 Index construction. In the following the index is validated stepwise in several multilevel models with self-rated health as the dependent variable and known control variables like sport (whereby endogeneity problems arise in the cross-section: Does more exercise lead to better health, or do healthier people exercise more?), sex, age and education. Confidence in the health care system is included as an independent variable. (The fact that these are two different dependent variables will be explained in more detail later).

### Hypotheses

This paper focuses on index construction and the related theoretical background. For subsequent quality control, the index is included in various multilevel models. Multilevel models are commonly used to control for country differences [8]. If the index in the multilevel model is able to reduce the variance between countries, it suggests that the index is measuring the right factors to control for country differences at a given level. Since the index should be able to control for country differences, I assume that the variance between countries (inter class correlation—ICC) is reduced with respect to self-rated health, which leads to my first hypothesis:

**H1.**  *If the index is included, the variance of self-rated health between countries will be reduced.*

The relevance of a variable can be shown, among other things, by how much variability of the dependent variable it can explain in a regression model [9]. Therefore, the index is valuable if including it into the model adds to explaining variability (R${}^{2}$), which leads to the second hypothesis:

**H2.**  *Including the index will increase the amount of variability of self-rated health.*

Personal or supplementary insurance policies can be taken out for which people pay themselves and thus invest in their own protection. In contrast, people can also have only basic security through their state. This means that the respective country invests. In other words, there are three outcomes: (a) people with no health insurance at all; (b) people with insurance through their country of residency; or/and (c) people who additionally insure themselves. Not every country has the same basic health care coverage.

The last hypothesis relates directly to the constructed index. The higher the values, the more people should be able to benefit from the health care system in their country. Low index values indicate that there is lower access to the health care system, while high values indicate that there is good access to the health care system. This means that a positive influence is assumed:

**H3.**  *The higher the index value, the better the self-rated health.*

In the following section on data base and methods, I will proceed to explain the data sets, the indicators and variables that were used and their connections. Afterwards, I will present the methods which I have applied. In doing so I will explain the theoretical background behind each method step-wise. Lastly, the results will be explained and discussed.

## 2. Data Base and Methods

### 2.1. Datasets

The dataset used here is the International Social Survey Programme (ISSP): Health and Health Care—ISSP 2011, founded in 1984 and currently compassing of 42 member states worldwide [10]. The survey takes place annually and has changing focus topics. So far, 2011 is the only survey wave with a focus on health and health care. Therefore cross-sectional data are available for the study. Between February 2011 and April 2013, a total of 55.081 people from 32 countries were interviewed. The data set is used because this is the only one that includes over 30 countries and additionally asks about self-rated health and confidence in the health care system.

In addition, the ISSP—Health and Health Care is complemented with macro health care criteria from the Global Health Expenditure Database (GHED) as collected by the World Health Organization [11]. The Global Health Expenditure Database includes 275 indicators from 192 countries over 20 years. It is updated annually with a two-year lag, and the data are collected by member states or estimated by WHO in case of gaps [11]. The additional process-generated data allows for combining individual assessment and objective macro criteria of health care systems for international comparison. Many GHED indicators are available in different units, which is why the number of 275 indicators sharply decreases when it comes to analysis. Likewise, not all indicators are available for the available countries of the ISSP 2011 and the corresponding year, which is why in sum 52 indicators are included in the analysis. This is only health care expenditure, as all other macro indicators, such as the availability of hospital beds, are not available.

One country (Taiwan) is excluded because the GHED dataset does not provide any data for the year 2011. Therefore all in all a total of 31 countries are included in the international comparison: Australia, Belgium, Bulgaria, Chile, China, Croatia, Czech Republic, Denmark, Finland, France, Germany, Great Britain, Israel, Italy, Japan, (South) Korea, Lithuania, Netherlands, Norway, Philippines, Poland, Portugal, Russia, Slovak, Slovenia, Spain, Sweden, South Africa, Switzerland, Turkey, and Middle Atlantic (United States of America).

### 2.2. Objective Macro Indicators

Since this is a country comparison, the analysis is not based on absolute values, but rather relative measures. The main relation refers to percentage of gross domestic product (GDP) spend in each country. It is used to measure current health expenditure and health capital expenditure in health care systems as well as general government expenditure and all health expenditure data. The gross domestic product is recorded as U.S. dollars per capita. Then, current health expenditure is mostly the reference for all indicators, as shown in Table 1. Government subsidy to social health insurance and self-employed contributions to social health insurance provide the reference to percentage of social health insurance. If it had been possible, only one unit would serve as reference (percent of GDP). Since this was not possible, the references were chosen so that they are, as far as possible, the same or do not vary too often. The most common reference is gross domestic product, followed first by current health expenditures and second by social health insurance.

Each indicator is assigned a weight to predict the impact of self-rated health on confidence in the health care system. In the end all indicators are included in the index, except countries and gross domestic product. They are included for reference and control reasons in estimating the weight for constructing the index and play no role in creating the index. However, the country variable is important for validating the index, as it is implemented as a second level control in the multilevel models.

### 2.3. Methods

A chronological explanation of the methods is given, with index construction being the focus of this work. First, missing data are imputed for macro indicators (GHED subset), using the R package “missForest” [12]. Then two steps are taken for furthering my approach: First, a data-driven procedure, called causal forest, is used to identify the individual’s confidence in their respective health care systems, with regards to the available objective macro criteria of health care systems. Afterwards, a weighted index is formed.

In a second step, the created health care index is validated in stepwise multilevel models with self-rated health being the dependent variable. There are two different models: A first one to construct the index and a second one to validate the index. Confidence in the health care system is the dependent variable to form the index, as it is the proxy for access to the health care system [13,14]. The dependent variable used to validate the index is self-rated health. Two different dependent variables are intentionally used to avoid tautological inferences. Further reasons for this decision are explained in Section 2.3.2 Index construction.

#### 2.3.1. Imputation Algorithm “missForest” for GHED Subset

The algorithm ’missForest’ is used to impute missing values particularly in the case of mixed-type data. The advantage of this imputation method is that categorical and continuous variables are considered at once and that it therefore does not ignore possible relations between different variable types including complex interactions and nonlinear relations [12]. Although most of the variables used have a continuous structure, it is of high relevance to control for the country level as well.

The basic idea is to quickly replace missing data and then iteratively improve the missing imputation using proximity. The fraction of trees for which a pair of observations shares a terminal node gives the approximation matrix and therefore explicitly uses the class label. First, a training set is drawn, which replaces the missing values with the average value. This is repeated until the algorithm is satisfied. Then a random forest is trained by using the imputed values computed so far. In the next step the approximation matrix is calculated and proximity is used as a weight for imputing missing values as a weighted average of the non-missing values [12].

The implemented out-of-bag (OOB) imputation error indicates the strength of the imputation error. The “missForest” algorithm shows two OOB imputation errors, one for categorial (PFC— proportion of falsely classified entries) and one for continuous variables (NRMSE—normalized root mean squared error). The value can be between zero and one and is better when closer to the lower bound [12].

Given that the number of macro indicators should be as large as possible, the GHED subset will be imputed. The ISSP is not imputed to avoid errors in self-rated health values. These are so individual that imputation seems questionable here.

#### 2.3.2. Index Interpretation

Confidence in the health care system is the dependent variable for the index construction, because it better reflects access to health care systems. Many studies show that people who trust their health care system also think that their access is better [13,14]. As mentioned before it is of great interest to look at subjective and objective level of health care systems simultaneously and therefore find a way to combine them in the index.

Various macro indicators, all of which are related to health care expenditure in one way or another, are weighted by importance. The importance of these indicators is determined from the influence of self-rated health on confidence in the health care system. It is considered which indicator moderates the relationship and to what extent. All indicators are included in the model at the same time and therefore control each other. This means that although only health expenditure is considered, a subjective level is built in. Since health care expenditure now explains the subjective link between self-rated health and confidence in the health care system.

This approach may seem somewhat questionable. However, it is an attempt to connect the objective level (country macro criteria) with the subjective level (influence of self-rated health on confidence in the health care system). Variable importance then indicates the extent to which the objective macro criterion is relevant for people’s access to their health care system, which is measured through the subjective link with the previous justification that people who trust their health care system more also assume better access [13,14]. Afterwards it is incorporated into the index accordingly. The relevance is measured by the moderating strength of each macro indicator [15]. This means that although the index shown consists purely of data on health care expenditures, these are weighted in such a way that they ultimately represent access to the health care system. More details about the index construction can be found in the next chapter.

#### 2.3.3. Index Construction

**Figure 1:**
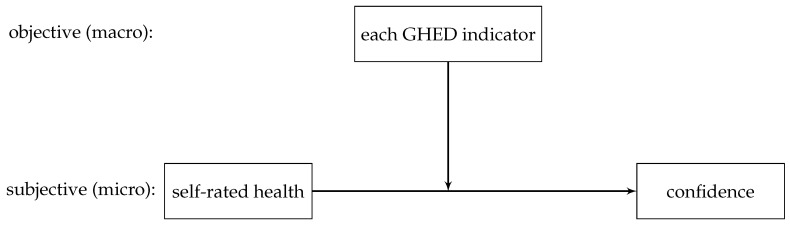
Link used for indicators weight; confidence = confidence in the health care system.

Implementing causal forests is a fairly new method that allows to calculate conditional average treatment effects [16]. It is likely that this method will soon become very popular in the social sciences, as it is the first data-driven method that is able to calculate estimators and confidence intervals, instead of the usual black box models. The technical details of the procedure are beyond the scope of this paper and can be found in the following source [16]. In this paper, causal forest is used to consider the variable importance and determine each indicators weight. The higher the variable importance, the more frequently the indicator moderates, which suggests that it has a broader influence and should therefore be weighted more highly [15]. For this, the independent variable (self-rated health) and the dependent variable (confidence in the health system) are fixed and all other indicators are included at once. Figure 1 presents the relationship, with which the variable importance for each indicator is determined:

To determine variable importance I considered confidence in the health care system and self-rated health to be quasi-metric, an approach which is necessary from a methodological point of view, because causal forest cannot include categorical dependent or independent variables [16]. Furthermore, the advantageous distribution of both variables and the loss of information this approach prevents in comparison to dichotomising the variables make it a reasonable choice.

Confidence in the health care system was measured with the following question: “In general, how much confidence do you have in the health care system in [your country]?” with response categories: none, very little, some, a great deal and complete confidence. Self-rated health was inquired by asking: In general, would you say your health is [excellent, very good, good, fair, poor, or can’t choose]? An index from 0 to 100 is formed to have a simple interpretation in percentage points, see Table 2.

#### 2.3.4. Multilevel Analysis for Validating the Constructed Index

In order to determine the correct weighting of the indicators for the index using causal forest (index construction), confidence in the health care system was used as the dependent variable [13,14]. The reason for this is that confidence in the health care system at the individual level best reflects access to the health care system. Those who trust their health care system will also assume that they can rely on it in the event of an emergency [17]. In the multilevel models the index is tested using self-rated health as the dependent variable. Many analyses rely on self-rated health as a measure because, unlike objective indicators, it is easy to survey and the correlation between objective and subjective health is very high [18]. Therefore, it is relevant whether the constructed index works in the context of the dependent variable self-rated health.

**Figure 2:**
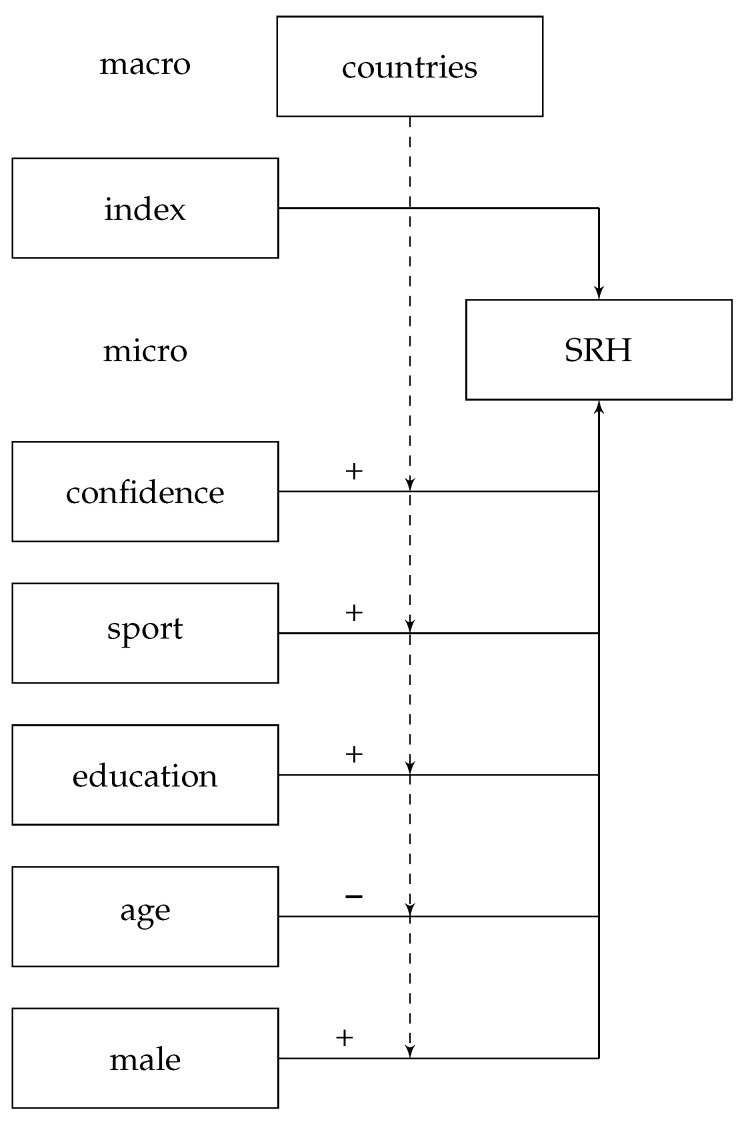
Full multilevel model for validating the created index. SRH = self-rated health, confidence = confidence in the health care system, dashed arrows indicate the potential influence through, for example, laws or regulations of countries at the micro level.

In order to control for potentially associations, several variables are included that show an impact on self-rated health: sport, education, sex and age as well as confidence in the health care system, see Figure 2.

Even if confidence in the healthcare system was the dependent variable for constructing the index, it serves as an independent variable when validating it. Including this variable into the model is supposed to prevent the index from reflecting just confidence in the healthcare system. Given the case that the index only reflects confidence in the healthcare system, it should lose its relevance after including confidence. Additionally, there is a significant and positive relationship between confidence in the health care system and self-rated health [19].

Physical activity does not only prevent physical problems. It can also be integrated in the treatment and rehabilitation of various diseases. People who are physically active on average consider themselves to be healthier and have fewer health problems [20]. Sports behaviour is surveyed with the question: How often do you exercise physically for at least 20 min so that you sweat or breathe more than usual? No sport at all is the reference, while the other outcomes are: once a month or less often, several times a month, several times a week and daily.

A low educational level is associated to a lower number of psychosocial resources [21,22], implying that educational background is positively associated to health outcomes [23]. Primary school is the reference, while the other five outcomes are: lower secondary, upper secondary, post secondary, non-tertiary, lower level tertiary, first stage and upper level tertiary.

Longitudinal studies show that self-rated health changes with age [24]. Physical health objectively deteriorates with age, but people tend to think that they are healthier than they are as they get older [25]. Age was queried openly and limited in the analysis to people between 18 and 82 years of age in order to define a uniform upper and lower limit (18 years of the actual possible characteristics) to reduce possible survival effects (distortions in favour of survivors) [26].

Men and women differ in their self-rated health and illness [27]. Based on the survey results, it is also important to consider the gender health paradox, which describes the observation that men consider themselves to be healthier than women, even though the latter have a higher life expectancy [28]. Sex was asked dichotomously by asking if one is a woman or a man. No alternative information could be given. Eleven people chose ’no information’ and are excluded from the analysis due to the small number of cases.

## 3. Results

### 3.1. Imputation Algorithm ’missForest’ for GHED Subset

The normalised root mean squared error (NRMSE) for continuous parts of the imputed dataset is 0.0005. This accounts for a very good performance [12]. The second value is the proportion of falsely classified entries (PFC) in the categorical part of the imputed data set [12], which in this case is zero, because there is no country missing and therefore no error can occur. Imputation errors are thus virtually excluded.

### 3.2. Variable Importance through Causal Forest

**Figure 3:**
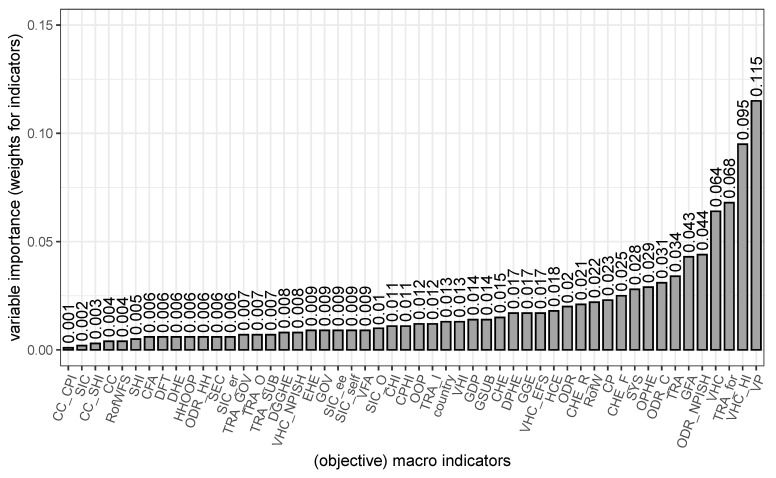
Variable importance of each macro indicator from the GHED subset. Causal forest with dependent variable confidence in the health care system and independent variable self-rated health. Meaning of macro indicator shortcuts see Table 1, N = 45.836.

Figure 3 shows variable importance (VI) for the influence of self-rated health (independent variable) on confidence in the health care system (dependent variable).

It seems that out of all 52 objective macro criteria voluntary prepayment (VI${}_{\mathrm{VP}}$ = 0.115) and voluntary health insurance schemes (VI${}_{\mathrm{VHC}\_\mathrm{HI}}$ = 0.095) have the strongest moderating relationship with the other variables. It appears that people, who are comfortable with providing adequate coverage and insurance for themselves, are statisfied with their health care system. Transfers distributed by government from a foreign origin (VI${}_{\mathrm{VHC}\_\mathrm{for}}$ = 0.068) is the third strongest influence factor and voluntary health care payment schemes come in fourth place (VI${}_{\mathrm{VHC}}$ = 0.064).

Variable importance cannot be interpreted in relation, but the scale still shows what is more, less or similarly relevant [15]. Compulsory private health insurance (VI${}_{\mathrm{CPHI}}$ = 0.011) and current health insurance (VI${}_{\mathrm{CHI}}$ = 0.011) for example are equally relevant.

Least important are compulsory private insurance schemes (VI${}_{\mathrm{CC}\_\mathrm{CPI}}$ = 0.001), social insurance contributions (VI${}_{\mathrm{SIC}}$ = 0.002) and social health insurance schemes (VI${}_{\mathrm{CC}\_\mathrm{SHI}}$ = 0.013), which seem to have almost no influence. The country of residency does seem to have a rather small influence (VI${}_{\mathrm{country}}$ = 0.013) as well as the gross domestic product (VI${}_{\mathrm{GDP}}$ = 0.014).

Although the variable importance is not to be interpreted as a relative measure, these variable importance values are used to weight the macro indicators for the index. Even though variable importance does not actually indicate weight, they determine how much an indicator moderates others. This shows the influence in a different way and is weighted accordingly. The country indicator as well as the gross domestic product are not included, since they were just included for control and reference reasons. Therefore, the additive index is constructed through this formula:

(1) $$index=\sum value_{\mathrm{variable}\;{\mathrm{importance}}_{i}}\times value_{{\mathrm{indicator}}_{i}}$$

Afterwards, the constructed index is limited to zero and 100 in order to allow easier interpretation through percentage points:

(2) $$index_{0-100}=\frac{value_{index}-min_{index}}{max_{index}-min_{index}}$$

Without the subjective level in the index, a value of of zero means that a country invests very little in their health care system and 100 means that the most is invested, regardless of who the investment actually comes from (people individually or country of residence). Weighting ensures that the subjective level, which depicts what people find most important (who invests how much), is taken into account in assessing how accessible they think their health care system is to them, as estimated by their confidence in the health care system. In other words, a low score means that people assume little access to the health care system, while a high score means that people assume good access.

### 3.3. Descriptive Statistics

Table 3 shows the descriptive statistics for the metric characteristics. For self-rated health, there is a value of zero if health is rated to be very bad and 100 if it is rated to be very good. The median of 50 and the mean of 52.41 show that the distribution is only minimally skewed to the left. In the index, a value of zero means that almost no access is assumed and 100 means that the access is very good. There is a left-skewed distribution, which means that more often less access is expected and less often more. Age is approximately normally distributed, averaging about 47 years with a standard deviation of about 16 years.

Table 4 shows the descriptive statistics of the categorical variables.

### 3.4. Bivariate

**Figure 4:**
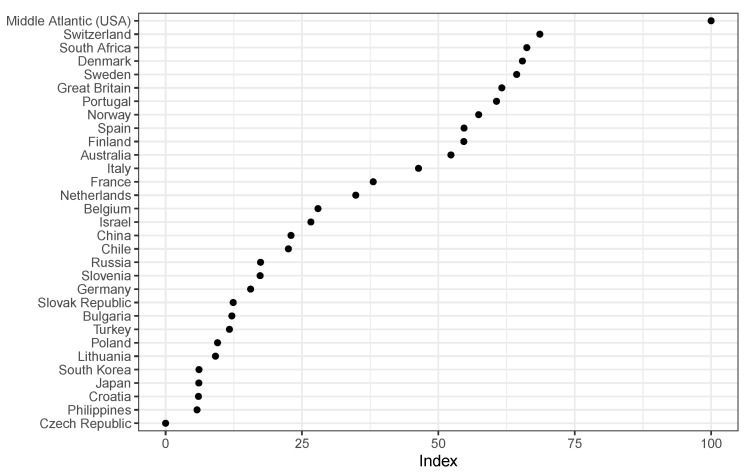
Link index and countries, N = 45.836.

Figure 4 shows the countries and the index in bivariate context. It indicates that the Czech Republic has the lowest index value (zero) and the Middle Atlantic has the highest (100). Switzerland has a similar value as South Africa, Denmark, Sweden, Great Britain and Portugal. Philippines, Croatia, Japan and South Korea have the lowest values after Czech Republic. As already mentioned in the descriptive statistics there are more countries, which expect less access in their health care systems (20 out of 31 countries).

The order is surprising in some respects and differs from other health care indices at the time, such as NUMBEO [29]. In the 2012 NUMBEO index, Japan ranks first, followed by Israel, Denmark, Sweden, Slovenia, Belgium, France and Switzerland. The weaker countries in terms of NUMBEO are Poland, China and Russia. A new order was to be expected in the new index, since the subjective level is included and not only objective macro indicators are compared. The approach shows a new arrangement of the countries, which needs to be validated.

### 3.5. Multilevel Models for Validating the Constructed Index

Health care systems can change, but this often takes several decades [30]. Therefore, fixed effects are assumed and a varying intercept and constant slope models are used. The multilevel analysis is performed with R version 4.0.0 by the package lme4 [31] in order to validate the index by using the hypotheses that have been established, see Table 5.

The full model explains about 15% of the variance in self-rated health. The control variables corroborate the influence that was assumed: An increase in physical activity comes with an increase in self-rated health (up to 7%), men assess their health to be better than women do (around 1.7%), the higher the educational level, the healthier one thinks to be (up to around 11%), and the older one is, the worse one estimates one’s own health to be (by around 4% per year). People who have more confidence in their health care system also consider themselves to be healthier (up to 13%). All control variables are statistically significant at the 1% level.

**H1.**  *If the index is included, the variance of self-rated health between countries will be reduced.*

In all models, the ICC decreases slightly after adding the index. This suggests that the index reduces differences between countries and therefore considers the appropriate factors on the macro level even with the weighting on the micro level link between confidence in the health care system on self-rated health. Additionally, the Akaike information criterion as well as the log-likelihood decreases slightly across the models. Therefore, it still measures macro indicators and is applicable for a between country comparison.

**H2.**  *Including the index will increase the amount of variability of self-rated health.*

In all models, the marginal $R^{2}$ increases slightly after adding the index, which implies that the index helps to explain more variability in the models. If we look at the full model, we see an increase of 0.9 percentage points, from 13.9% to 14.8%, which at first does not seem to be much, but in relative terms amounts to a marginal increase of 6.1%. The relative difference between the bivariate models is 35.7% or 1.0 percent point, from 1.8% to 2.8%.

**H3.**  *The higher the index value, the better the self-rated health.*

The estimates for the index are approximately equally strong and positive across all models (coefficients ranging from 0.078 to 0.105) and all are statistically significant at the 5% level. Which means that, as expected, the higher the index values, the better the self-rated health.

The added index does not change the influence of other estimates, which suggests that it adds in control and does not change the true effects of the other variables on the subjective level, especially confidence in the health care system. The index also does not lose its effect when confidence in the health care system is included. In addition, the following finding supports the index: $\tau_{country}$ decreases over all models after adding the index. This suggests that the deviation of countries from overall average decreases. There is also no change in deviation of individuals and countries, which is indicated by an equally large $\sigma^{2}$ within the models.

## 4. Discussion

An attempt was made to create a comprehensive index that can mitigate the previous ecological fallacy of inferring from the macro level to conditions on the micro level. For this purpose, the link between self-rated health and confidence in the health care system was examined at the micro level and the relevance of typical macro indicators was assessed on this basis. This was the first attempt of creating such an encompassing index which makes it difficult to compare it to similar research or put it in context. In the following, I will briefly discuss the approach, summarise the results, and discuss them individually.

The combination and weighting of the indicators was chosen by the algorithm exclusively. In terms of content, a fairly logical picture emerged: people who are self-insured are more satisfied with their health care system. All indicators were weighted and not only selected ones in order to discover all possible correlations. For further analysis, it would be useful to see if the same results can be achieved with fewer indicators (only the more relevant ones), so that individual index construction on different datasets can be facilitated and made accessible to everyone.

The goal would be to make it easy to create this index based on the dataset used, for example, let us say with the ten most relevant indicators. This presupposes that self-rated health as well as confidence in the health care system were surveyed. Self-rated health must be present in the data set in order to make subjective country comparisons for which this index is relevant. In the case of confidence in the health care system, trust in institutions could be used if necessary, although this still needs to be examined.

As a reminder, a low index score implies that very little access in health care systems is expected, while high scores suggest that a lot of access to health care systems is assumed. The index consists of weighted health care expenditures. The index does not differ if people individually or the state of residence spend money. However, it seems that individuals who spend more, whether through the government or personally, feel better about their health, which means that they have a better access to their health care system. Additionally, the index reduces country differences and increases the explained variance, even if just minimally. The connection between the objective macro level and the subjective micro level seems to be successful.

The index also corroborates that the average deviation of countries from the overall average decreases. This means that the countries become more similar and thus have less variance. At the same time, however, the variance of the individuals and the countries does not change and is thus constant, which suggests that the estimations for the individuals are robust, as one can see in Table 5. This is another advantage of the index; although it was constructed using confidence in the health care system, its inclusion does not change the estimates of the control variables or the estimates of confidence in the health care system.

To return to the example of the United States of America (U.S.): even after weighting health care expenditures at the individual level, they are at the top of the index. The U.S. is known to have very high private health care spending (similar to Switzerland), which even in highly developed countries does not necessarily indicate a good health care system. However, in this analysis, private spending was the determining factor of whether or not one trusted their health care system, which in turn was the proxy for access to the health care system. This seems reasonable, since people who self-insure also know the extent to which they are insured and their access to specific benefits. What gives the U.S. the first place is the excellent education in the field of medicine. People from all over the world, who can afford it, travel to the U.S. to get the best treatment from specialists.

In Figure 4, we see that countries from the Middle Atlantic receive the highest index value, followed by Switzerland, South Africa, Denmark, Sweden and the United Kingdom. In other health comparisons across countries, South Africa would have never been placed in this group [29], but perhaps South Africa is underestimated. In the COVID-19 pandemic, South Africa was even credited by the World Health Organization [32]. This is still a very interesting finding and should be investigated in detail in future studies. In this case, the countries with the worst access to the health care system are Japan, Croatia, Philippines and the Czech Republic. Croatia and the Czech Republic are ranked at a similarly low level by NUMBEO, with Japan and the Philippines coming in better. As explained above, these differences are to be expected due to the inclusion of the subjective level.

It would also be interesting to see to what extent the index can capture different types of insurance to include not only the macro level but also the meso level.

## 5. Conclusions

The new index combines the objective macro level and the subjective micro level and should measure the access to the health care system. In consideration of self-rated health, it reduces country differences and increases the explained variance. It also suggests that the better the access to the health system, the healthier people feel. This is the first step towards a more meaningful analysis for country comparisons. The inclusion of self-rated health provides a series of nuances that enrich the vision of the perception of health care systems, since it includes the vision of the perception of people’s health from different countries and cultures. Now it is important to keep at it and carry out further analyses in this regard, so that it will soon be possible to create such an index individually, for example, if one wants to compare countries in health issues on the subjective level.

## Figures and Tables

**Table 1:** GHED indicators with reference and shortcut used to create the index.

	Shortcut	Indicator
*indicators*		
1	CHE	current health expenditure (CHE) as % of gross domestic product (GDP)
2	HCE	health capital expenditure as % of GDP
3	DHE	domestic health expenditure as % of CHE
4	DGGHE	domestic general government health expenditure as % of CHE
5	DPHE	domestic private health expenditure as % of CHE
6	VHI	voluntary health insurance as % of CHE
7	OOP	out-of-pocket as % of CHE
8	OPHE	other private health expenditure as % of CHE
9	EHE	external health expenditure as % of CHE
10	CFA	compulsory financing arrangements as % of CHE
11	GFA	government financing arrangements as % of CHE
12	CHI	compulsory health insurance as % of CHE
13	SHI	social health insurance (SHI) as % of CHE
14	VFA	voluntary financing arragements as % of CHE
15	RofW	rest of the world as % of CHE
16	CPHI	compulsory private health insurance as % of CHE
17	GSUB	government subsidy to social health insurance as % of SHI
18	SEC	self-employed contributions to social health insurance as % of SHI
19	GGE	general government expenditure as % of GDP
*health expenditure data (all in % of GDP)—revenues*		
20	CHE_R	current health expenditure by revenues of health care financing schemes
21	TRA	transfers from government domestic revenue (allocated to health purposes)
22	TRA_I	international transfer and grants
23	TRA_GOV	transfers by government on behalf of specific groups
24	TRA_SUB	subsidies
25	TRA_O	other transfers from government domestic revenue
26	TRA_for	transfers distributed by government from foreign orgin
27	SIC	social insurance contribution
28	SIC_ee	social insurance contribution from employees
29	SIC_er	social insurance contribution from employers
30	SIC_self	social insurance contribution from employees
31	SIC_O	other social insurance contributions
32	CP	compulsory prepayment
33	VP	voluntary prepayment
34	ODR	other domestic revenues
35	ODR_HH	other revenues from households
36	ODR_C	other revenues from corporations
37	ODR_NPISH	other revenues from non-profit institutions serving households (NPISH)
38	DFE	direct foreign transfers
*health expenditure data (all in % of GDP)—financing schemes*		
39	CHE_F	current health expenditure by financing schemes
40	SYS	government schemes and compulsory contributory health care financing schemes
41	GOV	government schemes
42	CC	compulsory contributory health insurance schemes
43	CC_SHI	social health insurance schemes
44	CC_CPI	compulsory private insurance scheme
45	VHC	voluntary health care payment schemes
46	VHC_HI	voluntary health insurance schemes
47	VHC_NPISH	non-profit institutions serving households financing scheme
48	VHC_EFS	enterprise financing scheme
49	HHOOP	household out-of-pocket payment
50	RofWFS	rest of the world financing schemes (non-resident)
*general macro data*		
51	GDP	gross domestic product in US dollar per capita
52	country	countries from ISSP 2011

**Table 2:** Distribution of self-rated health and confidence in the health care system before and after recoding.

**original scale**	1	2	3	4	5
**new scale (%)**	0%	25%	50%	75%	100%
**N** ${}_{\mathrm{confidence}}$	2.655	7.293	17.399	15.192	3.297
**N** ${}_{\mathrm{self}-\mathrm{rated}\;\mathrm{health}}$	2.277	10.141	18.147	11.431	3.840

N = 48.836, confidence = confidence in the health care system.

**Table 3:** Description of metric variables.

Variable	N	Min	Max	$\tilde{x}$	$\bar{x}$	S
SRH	45.836	0	100	50.00	52.41	24.94
index	45.836	0	100	27.94	34.88	24.51
age	45.836	18	82	46.00	46.77	16.29

SRH = self-rated health.

**Table 4:** Description of categorial variables, N = 45.836.

Variable	Percentage	N
**confidence health care system**		
*none*	5.79%	2.655
*very little*	15.91%	7.293
*some*	37.97%	17.399
*a great deal*	33.14%	15.192
*complete*	7.19%	3.297
**sport**		
*never*	24.01%	11.004
*once a month or less*	14.63%	6.704
*several times a month*	20.10%	9.213
*several times a week*	26.09%	11.960
*daily*	15.17%	6.955
**sex**		
*female*	54.35%	24.911
*male*	45.65%	20.925
**education**		
*primary*	9.73%	4.461
*lower secondary*	24.99%	11.456
*upper secondary*	25.43%	11.654
*post secondary*	12.76%	5.848
*lower level tertiary*	16.04%	7.351
*upper level teritary*	11.05%	5.066

**Table 5:** Varying intercept constant slope model: estimation (standard error) [95% confidence interval].

Dependent Variable:	Intercept Only Model (ICO)		Bivariate Model		Multivariate Models	
SRH (0–100%)	ICO	ICO + Index	Bivariate	Bivariate + Index	Multivariate	Multivariate + Index
**index**		0.105 * (0.044) [0.020, 0.191]		0.096 * (0.040) [0.018, 0.175]		0.078 * (0.037) [0.005, 0.150]
**confidence**						
reference: none						
- very little			2.643 ** (0.550) [1.561, 3.716]	2.639 ** (0.550) [1.561, 3.716]	2.232 ** (0.515) [1.223, 3.241]	2.228 ** (0.515) [1.219, 3.237]
- some			5.517 ** (0.515) [4.509, 6.526]	5.513 ** (0.515) [4.504, 6.521]	4.892 ** (0.482) [3.947, 5.837]	4.888 ** (0.482) [3.943, 5.833]
- a great deal			9.867 ** (0.528) [8.832, 10.902]	9.860 ** (0.528) [8.825, 10.895]	9.305 ** (0.495) [8.335, 10.276]	9.298 ** (0.495) [8.328, 10.269]
- complete			12.142 ** (0.643) [10.881, 13.403]	12.140 ** (0.643) [10.879, 13.401]	12.645 ** (0.603) [11.464, 13.827]	12.643 ** (0.603) [11.461, 13.825]
**sport**						
reference: never						
- once a month or less					1.017 ** (0.357) [0.317, 1.716]	1.015 ** (0.357) [−0.315, 1.714]
- several times month					3.526 ** (0.334) [2.872, 4.180]	3.521 ** (0.334) [2.867, 4.175]
- several times week					7.497 ** (0.320) [6.868, 8.125]	7.488 ** (0.320) [6.859, 8.115]
- daily					7.447 ** (0.356) [6.749, 8.145]	7.441 ** (0.356) [6.753, 8.115]
**sex**						
reference: female						
- male					1.694 ** (0.212) [1.278, 2.110]	1.695 ** (0.212) [1.279, 2.111]
**education**						
reference: primary						
- lower secondary					2.385 ** (0.426) [1.550, 3.220]	2.390 ** (0.426) [1.1556, 3.225]
- upper secondary					5.252 ** (0.438) [4.409, 6.417]	5.262 ** (0.438) [4.403, 6.120]
- post secondary					5.415 ** (0.507) [4.394, 6.111]	5.412 ** (0.507) [4.419, 6.404]
- lower level tertiary					7.989 ** (0.472) [7.064, 8.915]	7.990 ** (0.472) [7.065, 8.915]
- upper level tertiary					11.075 ** (0.511) [10.073, 12.077]	11.073 ** (0.511) [10.071, 12.075]
**age**					−3.997 ** (0.069) [−4.132, −3.862]	−3.825 ** (0.081) [-3.984, -3.666]
intercept	52.140 ** (1.194) [49.800, 54.481]	48.555 ** (1.850) [44.930, 52.180]	45.663 ** (1.191) [43.328, 47.998]	42.394 ** (1.761) [38.942, 45.846]	35.876 ** (1.158) [33.607, 38.145]	33.241 ** (1.674) [29.959, 36.523]
$\sigma^{2}$	584.91	584.91	575.21	575.21	503.93	503.93
$\tau_{country}$	43.75	37.64	36.88	31.81	30.04	26.92
ICC	0.070	0.060	0.060	0.050	0.060	0.050
conditional $R^{2}$	0.070	0.070	0.077	0.079	0.187	0.191
marginal $R^{2}$	0.000	0.010	0.018	0.028	0.139	0.148
LL${}_{\mathrm{model}}$	−211,130.94	−211,128.09	−210,743.41	−210,740.59	−207,704.61	−207,702.41
AIC	422,267.88	422,264.17	421,500.81	421,497.19	415,445.21	415,442.81

* *p* < 0.05, ** *p* < 0.01, $N_{\mathrm{countries}}$ = 31, $N_{\mathrm{individidual}}$ = 45.836, $N_{\min}$ = 752, $N_{\max}$ = 4.125, $\tau_{\mathrm{countries}}$ = random effect variance of country level, LL = Log-Likelihood, AIC = Akaike information criterion, BIC = Bayesian information criterion.

## Data Availability

Data: The ISSP data can be accessed on the following homepage: http://www.issp.org/data-download (accessed on 31 January 2022). The data of the GHED can be downloaded here: https://apps.who.int/nha/database/Select/Indicators/en (accessed on 31 January 2022). I am happy to provide my data extension to the ISSP 2011 individually. Please contact me for more information.
